# The delivery of hsa-miR-11401 by extracellular vesicles can relieve doxorubicin-induced mesenchymal stem cell apoptosis

**DOI:** 10.1186/s13287-021-02156-5

**Published:** 2021-01-22

**Authors:** Huifang Li, Haoyan Huang, Xiaoniao Chen, Shang Chen, Lu Yu, Chen Wang, Yue Liu, Kaiyue Zhang, Lingling Wu, Zhong-Chao Han, Na Liu, Jie Wu, Zongjin Li

**Affiliations:** 1 Nankai University School of Medicine, Tianjin, China; 2 The Key Laboratory of Bioactive Materials, Ministry of Education, Nankai University, the College of Life Sciences, Tianjin, China; 3 Beijing Tongren Eye Center, Beijing Tongren Hospital, Capital Medical University, Beijing, 100730 China; 4 State Key Laboratory of Kidney Diseases, Chinese PLA General Hospital, Beijing, 100853 China; 5 Jiangxi Engineering Research Center for Stem Cell, Shangrao, Jiangxi China; 6 Tianjin Key Laboratory of Engineering Technologies for Cell Pharmaceutical, National Engineering Research Center of Cell Products, AmCellGene Co., Ltd., Tianjin, China; 7 Beijing Engineering Laboratory of Perinatal Stem Cells, Beijing Institute of Health and Stem Cells, Health & Biotech Co, Beijing, China

**Keywords:** Doxorubicin, Mesenchymal stem cells (MSCs), Extracellular vesicles (EVs), Hsa-miR-11401

## Abstract

**Background:**

Chemotherapy is an effective anti-tumor treatment. Mesenchymal stem cells (MSCs), exerting therapy effect on injured tissues during chemotherapy, may be damaged in the process. The possibility of self-healing through long-range paracrine and the mechanisms are unclear.

**Methods:**

Doxorubicin, a commonly used chemotherapy drug, was to treat human umbilical cord-derived mesenchymal stem cells (hUC-MSCs) for 6 h as an in vitro cell model of chemotherapy-induced damage. Then we use extracellular vesicles derived from placental mesenchymal stem cells (hP-MSCs) to investigate the therapeutic potential of MSCs-EVs for chemotherapy injury. The mechanism was explored using microRNA sequencing.

**Results:**

MSC-derived extracellular vesicles significantly alleviated the chemotherapy-induced apoptosis. Using microRNA sequencing, we identified hsa-miR-11401, which was downregulated in the Dox group but upregulated in the EV group. The upregulation of hsa-miR-11401 reduced the expression of SCOTIN, thereby inhibiting p53-dependent cell apoptosis.

**Conclusions:**

Hsa-miR-11401 expressed by MSCs can be transported to chemotherapy-damaged cells by EVs, reducing the high expression of SCOTIN in damaged cells, thereby inhibiting SCOTIN-mediated apoptosis.

**Supplementary Information:**

The online version contains supplementary material available at 10.1186/s13287-021-02156-5.

## Background

Chemotherapy is an effective curative method for cancers. While the drugs during chemotherapy work not just on the cancer cells, it may inevitably affect the state of part somatic cells. The cytotoxicity induced by the nonspecific mechanism and duration of medication is nonnegligible. Increasing evidence indicated the adverse effect severely impacted the life quality of cancer patients and lead to poor prognosis. The broad spectrum anticancer agent, doxorubicin (Dox), showed excellent anticancer effects for various cancers, including hematological malignancies and others sarcomas [1–3]. But the complications induced by doxorubicin cytotoxicity limited its wide application. The relative studies about doxorubicin cytotoxicity were generally reported [4, 5]. Therefore, there is a higher requirement for the implement of chemotherapeutic agents to avoid chemotherapy cytotoxicity. The exploration of therapeutic measures has been in progress.

Mesenchymal stem cells (MSCs), a type of adult stem cell, can regulate many vital life activities and are indispensable for the repair and regeneration of certain injured tissues [6–9]. Its therapeutic effects can be achieved by replacing damaged cells or by its paracrine. Some researchers have found that MSCs can alleviate cell apoptosis, and that can be used to treat doxorubicin-induced cytotoxicity in basic researches [10, 11]. These findings indicated that mesenchymal stem cells may play an important role to alleviate chemotherapy injury. Therefore, it is of great significance to maintain the viability and functional state of mesenchymal stem cells during chemotherapy. However, MSCs may also be damaged in this process and its therapy effects may be abolished. Therefore, how MSCs respond to chemotherapeutic agents and the fate of MSCs during chemotherapy are not clear.

Extracellular vesicles (EVs) derived from MSCs, mediating cell-to-cell communication, can be used as substitute for MSCs in the field of regenerative medicine and damage repair [12]. This indicated that one of the critical factors in the paracrine mechanism of MSCs is extracellular vesicles. Its lipid membrane structure endows it the property of performing biological functions without immunogenicity, a shortcoming of stem cell therapy. The cytokines and chemokines in EVs are the main regulators of cell homeostasis. Moreover, it is reported that extracellular vesicles derived from MSCs have anti-apoptosis effects, which is related to these cell growth factors in EVs. In addition, the MSCs and their extracellular vesicles have been applied for the therapy of chemotherapy injury in relative studies [10], which indicated that MSCs own the protective effect against doxorubicin. The extracellular vesicles derived from embryonic stem cells also reported to significantly alleviate the inflammation and apoptosis induced by doxorubicin [13], which implied the potential that stem cells may be repaired through long-range paracrine during chemotherapy in vivo. Therefore, we want to know whether the injured MSCs induced by chemotherapy agents are cured by extracellular vesicles of MSCs from other source and explore the mechanism of MSC self-healing during chemotherapy in this way. For the consideration that MSCs from one source may be damaged at the same degree, the function of which may be broken, we chose extracellular vesicles of MSCs from another source for the therapy of damaged MSCs. In our study, based on the remarkable cytotoxicity of doxorubicin, we constructed an in vitro doxorubicin cytotoxicity model and investigated the anti-cytotoxicity effects of extracellular vesicles derived from MSCs. We next employed the miRNA sequencing to find the effective factors in EVs against cytotoxicity induced by doxorubicin.

## Methods

### Cell culture

The human placental mesenchymal stem cells (hP-MSCs) and human umbilical cord-derived mesenchymal stem cells (hUC-MSCs) used in this study were from the preserved cells in our lab, which were isolated from the same source as that used in a previous study [14]. And the hP-MSCs and hUC-MSCs were isolated according to the methods introduced in relative studies [14, 15]. Briefly, the tissues were all cut into pieces and digested in the aid of collagenase II (Gibco, Grand Island, NY, USA) and trypsin (Gibco). The hP-MSCs were obtained from following steps. Tissue fragments were digested in collagenase II at 37 °C for 1 h and trypsin at 37 °C for 30 min respectively. About the hUC-MSCs, the tissue fragments were also digested in collagenase II at 37 °C for 30 min and trypsin at 37 °C for 30 min respectively. The collected cells were seeded into T75 flasks (Corning, Corning, NY, USA) and cultured in Dulbecco’s modified Eagle’s medium (DMEM)/F12 medium with 10% fetal bovine serum (FBS; HyClone, Logan, UT) and 100 U/mL penicillin-streptomycin (Gibco). To separate the extracellular vesicles in serum prior to application, the FBS for the culture of hP-MSCs was ultracentrifuged at 100,000×*g* overnight, the sediment of which was discarded. Then, the supernatant was filtered through a 0.22-μm membrane. The operations were completed under aseptic conditions. The hP-MSCs and hUC-MSCs used in subsequent experiments were passages 8 and 10. They were negative from mycoplasma tests and were maintained in a humidified incubator with 5% CO_2_ at 37 °C.

### Isolation of EVs from MSCs

Purified EVs were isolated and yielded from the supernatant of hP-MSC through differential centrifugation as previously described [16]. Briefly, after 2 days of culture in the Dulbecco’s modified Eagle’s medium (DMEM)/F12 medium (Gibco, Grand Island, NY), the conditioned medium of hP-MSC was centrifuged at 500*g* for 5 min and at 12,000*g* for 20 min, which were to remove cell debris and apoptotic bodies, respectively. Then, the ultracentrifugation of 100,000*g* for 70 min at 4 °C was operated to isolate EVs. The harvested EVs were resuspended in phosphate-buffered saline (PBS), followed by a second ultracentrifugation to discard contaminative proteins.

### Characterization of MSC-derived EVs

Transmission electron microscopy (TEM; HT7700, Hitachi, Japan) was used to observe the morphology of isolated EVs. A drop of EVs (20 μl) was subjected to a carbon film (Zhongjingkeyi Technology, Beijing, China) to incubate 5 min at room temperature, the excess liquid of which was removed with filter paper. Stained with 2% uranyl acetate for 30 s, the specimen was served as negative control. All samples being air-dried were imaged with TEM. The particle size of isolated EVs was determined via dynamic light scattering (DLS). And the protein concentration was quantitated using a BCA Protein Assay Kit (Promega, Madison, Wisconsin).

### Western blotting analysis

The samples used for identification of widely expressed protein markers in EVs and quantification of caspase 3 and cleaved-caspase 3 expression in three groups (Blank, Dox, EV) were suspended in 100 μl radio-immunoprecipitation assay (RIPA) buffer (Solarbio, Shanghai, China), the protein of which (30 μg) was subjected to 10% SDS-PAGE and transferred to polyvinylidene fluoride (PVDF) membranes (Millipore, Darmstadt, Germany). After being immersed in 5% nonfat milk for 2 h, they were incubated with primary antibodies overnight at 4 °C and secondary antibodies for 2 h at room temperature. The signal was detected by the Pierce enhanced chemiluminescence western blotting substrate (Millipore). The primary antibodies used for western blotting analysis were as follows: rabbit anti-Alix (Wanleibio, Shengyang, China), rabbit anti-CD9 (Abcam, Cambridge, UK), rabbit anti-CD63 (Wanleibio, Shengyang, China), rabbit anti-TSG101 (Abcam, Cambridge, UK), rabbit anti-caspase 3 (Wanleibio, Shengyang, China), rabbit anti-cleaved-caspase 3 (Wanleibio, Shengyang, China), and mouse anti-tubulin (Abcam, Cambridge, UK). Relevant experimental operations were following the manufacturer’s instructions.

### In vitro internalization of Dil-labeled EVs

To verify EVs can be internalized by hUC-MSCs, CM-DiI (Invitrogen)-labeled EVs were used to visualize its distribution in hUC-MSCs. According to the manufacturer’s protocol, the mixture of EVs and 1 mmol/L CM-DiI was co-incubated for 5 min at room temperature. Excess dye was removed through ultracentrifugation at 100,000×*g* for 70 min at 4 °C. Then, the labeled EVs were resuspended in PBS. Finally, CM-DiI-labeled EVs were incubated with hUC-MSCs at 37 °C for 6 h, after which the hUC-MSCs were fixed in 4% paraformaldehyde. The labeled EVs were observed under a fluorescent microscope (Nikon) and quantified on the basis of fluorescence density.

### Hoechst 33342 staining

To evaluate the anti-apoptosis property of extracellular vesicles from hP-MSCs, the Hoechst 33342 (MedChem Express) was used to distinguish the apoptotic cells in the three different groups (Blank, Dox, EV). 3 × 10^5^/well hUC-MSCs were seeded into a 6-well plate. With doxorubicin treatment for 6 h and extracellular vesicle therapy for 24 h being completed, Hoechst 33342 was used to stain cells for 10 min. Under a fluorescence microscope (Nikon), the apoptotic cells were recognized as bright blue nuclei.

### Cell viability analysis

A cell counting kit-8 (CCK-8) (MedChem Express, Monmouth Junction, NJ) was used to assess the viability of hUC-MSCs treated with doxorubicin. According to the manufacturer’s protocol, hUC-MSCs were seeded into a 96-well plate and treated with doxorubicin for 6 h, after which basic culture medium containing 10% CCK-8 solution was added into every well. The mixture was incubated for 3 h at 37 °C. Survival rate reflected by absorbance value at 450 nm was determined by using a microplate reader (Promega).

### RNA isolation and real-time PCR analysis

In this study, the mRNA expression of apoptosis-related factors, caspase 9, caspase 3, and bax, were detected through real-time PCR. Meanwhile, we also verified the expression of hsa-miR-11401 and its target gene, SCOTIN. As reported, the RNA was extracted from samples in lysate of TRIzol (Invitrogen, Grand Island, NY) as described previously. Subsequently, RNA was reversely transcribed into cDNA. The expressions of interest genes were detected by using a SYBR green-based real-time detection method and expressed as 2^(−ΔΔCT)^. The sequences of primers used in this study are showed in Table 1 (Additional file 1).

### Flow cytometric analysis

The hUC-MSCs were cultured in a 6-well plate at a density of 3 × 10^5^/well. With doxorubicin treating for 6 h and EVs remedying for 24 h, hUC-MSCs were digested into single cells. Then, these cells were stained with Annexin V-FITC and PI for characterization of early and late apoptotic cells respectively, which were for further flow cytometry analysis. The single-stained hUC-MSCs with Annexin V-FITC or PI were treated as a negative control group respectively.

### Immunofluorescence staining

Immunofluorescence staining was adopted to visualize the expression of cleaved-caspase 3. Briefly, hUC-MSCs of different treatment groups were fixed in cold acetone for 10 min, permeated in 0.1% Triton-PBS for 10 min, and blocked in 5% normal goat serum (Beyotime, C0265) for 1 h at 4 °C. Subsequently, the samples were incubated with primary antibody against cleaved-caspase3 overnight at 4 °C and appropriate secondary antibodies for 2 h at room temperature. A mounting solution containing 4,6-diamidino-2-phenylindole (DAPI, Vector Laboratories, Burlingame, CA) was used to counterstain nuclei for 5 min. With every operation being performed except for blocking, 0.01 mM PBS was used to rinse excess solution. Finally, images were observed under a fluorescence microscope (Olympus), and the expression of interest protein was analyzed by ImageJ software.

### Doxorubicin accumulation assay

The hUC-MSCs seeded into 24-well plates were treated with doxorubicin for different periods, after which 4% paraformaldehyde was used to fix these samples for 15 min at room temperature. And the nucleus were stained with 4,6-diamidino-2-phenylindole (DAPI; Vector Laboratories, Burlingame, CA) to detect their locations and to assure they are almost identical intensity in every well. Then, the intracellular fluorescent signals of doxorubicin were visualized under the fluorescence microscope (Olympus) at excitation/emission wavelengths of 488/550 nm.

### MicroRNA-inhibitor transfect

After the cell intensity growing to 80% confluence, 100 nM hsa-miR-11401 inhibitor (RiboBio, Guangzhou, China) was transfected into hP-MSCs with the aid of Lipofectamine 2000 in Opti MEM (Invitrogen, Grand Island, NY, USA). With inhibitor transfecting for 6 h, the cells were cultured in DMEM/F12 medium containing EV-free FBS for 48 h. The extracellular vesicles containing hsa-miR-11401 inhibitor were collected from cell supernatant of transfected hP-MSCs.

### MicroRNA sequencing

The RNA harvested from samples were qualificated in different methods, the purity, and integrity of which were checked by a NanoPhotometer® spectrophotometer (IMPLEN, CA, USA) and the RNA Nano 6000 Assay Kit of the Agilent Bioanalyzer 2100 system (Agilent Technologies, CA, USA), respectively. And the concentration was determined by Qubit® RNA Assay Kit in a Qubit® 2.0 Flurometer (Life Technologies, CA, USA).

Three-microgram RNA per sample was used as input material for the small RNA library. NEBNext® Multiplex Small RNA Library Prep Set for Illumina® (NEB, USA.) was used for the generation of sequencing libraries, and index codes were added to attribute sequences to each sample. According to the manufacturer’s recommendations, 3′ adaptors were firstly ligated to 3′ end of small RNA and excess 3′ SR adaptors were hybridized to the SR RT Primer. Then, 5′ adaptors were followed to ligate to the 5′ ends of small RNA. With 3′ and 5′ adaptor being successfully ligated to small RNA, they were reversely transformed to a single strand of cDNA, which were amplified by LongAmp Taq 2X Master Mix, SR Primer for Illumina and index primer. PCR products were purified on an 8% polyacrylamide gel (100 V, 80 min). Purified DNA fragments corresponding to 140~160 bp of the PCR products were recovered and dissolved in 8 μl elution buffer. DNA High Sensitivity Chips were finally used to assess the quality of library on the Agilent Bioanalyzer 2100 system.

The clustering of the index-coded sample was performed on a cBot Cluster Generation System using TruSeq SR Cluster Kit v3-cBot-HS (Illumina). With cluster completed, the library preparations were sequenced on Illumina Hiseq 2500/2000 platform, from which 50 bp single cells and reads were generated.

### Bioinformatics analysis

Qualified data were mapped to the reference sequence for recognition and annotation of known small RNA, which were used for further analysis, including target gene prediction, differential expression of miRNA, and GO and KEGG enrichment analysis. Unmapped miRNA was for prediction of novel miRNA.

### Statistical analysis

All results presented in this study were obtained from three independent experiments for each condition and analyzed by GraphPad. Relative data was expressed as standard error of the mean (SEM). Differences were considered statistically significant at *P* < 0.05.

## Results

### Doxorubicin-induced cell apoptosis in hUC-MSCs

**Fig. 1:**
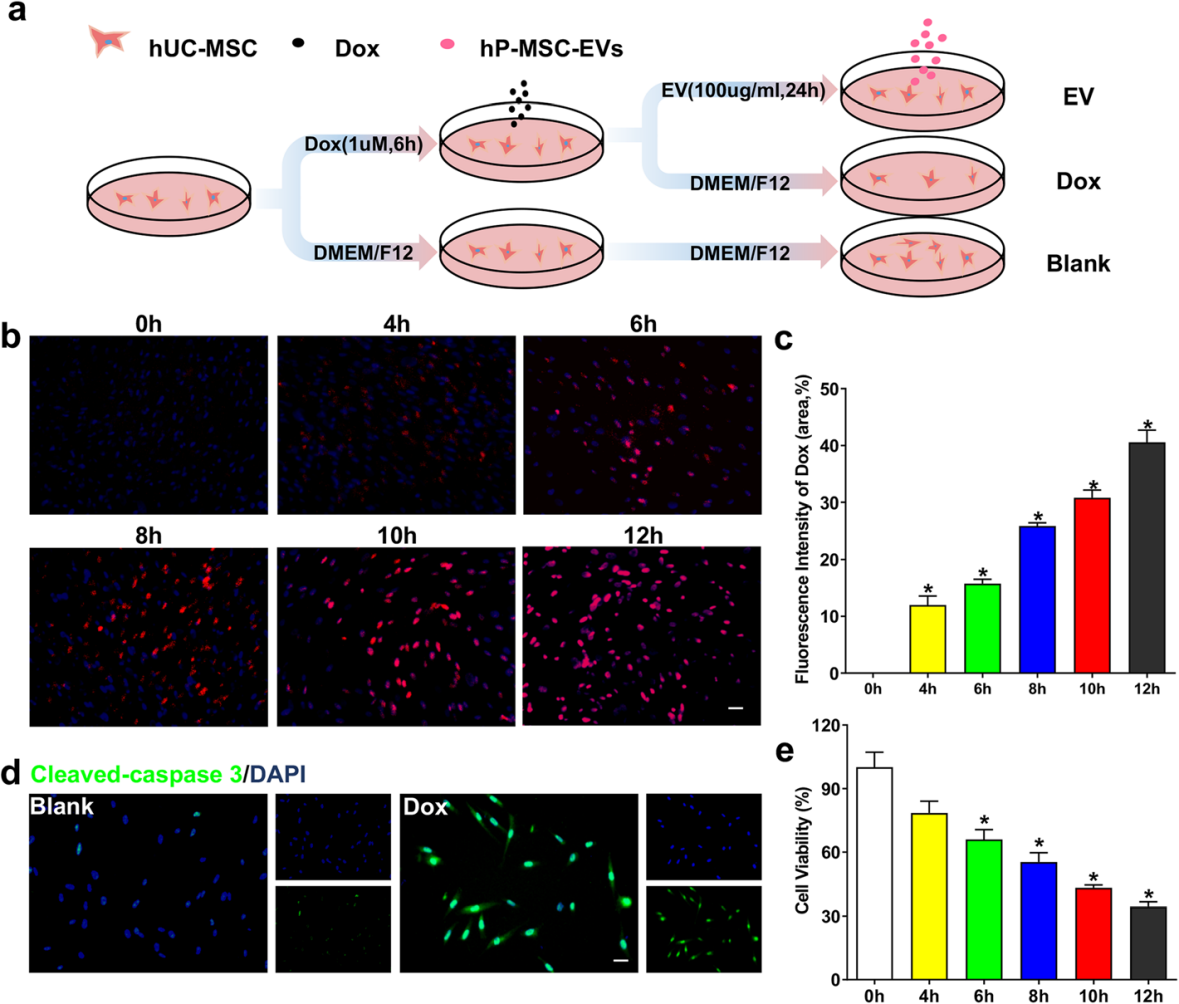
Dox induced cell apoptosis in hUC-MSCs. **a** The workflow of doxorubicin cytotoxicity model in vitro. **b** Representative immunofluorescence images of hUC-MSCs treated with doxorubicin for 4, 6, 8, 10, and 12 h. Normal hUC-MSCs were taken as control group. Intracellular accumulation of doxorubicin, revealed by the red fluorescence, was increasing with treatment period being prolonged. Scale bars, 20 μm. **c** Quantification of doxorubicin in hUC-MSCs of incremental treatment period, which showed consistent trend with immunofluorescence image. *n* = 3, ^*^P < 0.05 versus Blank groups. **d** Representative immunofluorescence images of stained cleaved-caspase 3 (green) in hUC-MSCs with doxorubicin treating for 6 h. Cell nucleus were also stained with DAPI (blue) for colocalization. Scale bars, 20 μm. **e** The cell survival rate of hUC-MSCs analyzed by CCK-8 was decreasing in a time-dependent manner. *n* = 3, ^*^P < 0.05 versus Blank groups

As an anthracycline anticancer drug, doxorubicin exerted apparent curative effect for numerous tumors, yet the cytotoxicity of which on somatic cells is also very severe [1–5]. In order to investigate the anti-cytotoxicity effect of MSC-EVs, we firstly established the in vitro cytotoxicity model using hUC-MSCs induced by doxorubicin (Fig. 1a). Previous studies revealed that the apoptosis of targeted cell treated with doxorubicin was related to its intracellular accumulation [17], which can be visualized through fluorescent signal of doxorubicin. In our study, hUC-MSCs were incubated with 1 μM doxorubicin for different periods of 4, 6, 8, 10, and 12 h. Based on the fluorescence images of hUC-MSCs, with processing time being prolonged, the intracellular doxorubicin, emitting red fluorescence at 488/550 nm, was increased incrementally (Fig. 1b). This part of results presented that fluorescent signal was positively correlated with the incubation period, which is consistent with fluorescent intensity (Fig. 1c). Subsequently, cleaved-caspase3, a marker of cell apoptosis, was increased in doxorubicin-induced hUC-MSCs, compared to blank groups (Fig. 1d). A CCK-8 cell viability analysis further provided definite evidence. As expected, against the increasing trend of fluorescent signal, the survival rate of hUC-MSCs was gradually declining (Fig. 1e). All above results demonstrated that doxorubicin can be internalized into hUC-MSCs to play a role of inducing apoptosis. Furthermore, with the periods of doxorubicin working on hUC-MSCs increasing, intracellular fluorescent signal is more robust, which demonstrated its rich accumulation. Concurrently, the targeted cells presented lower viability.

### Characterization of hP-MSC-derived EVs

**Fig. 2:**
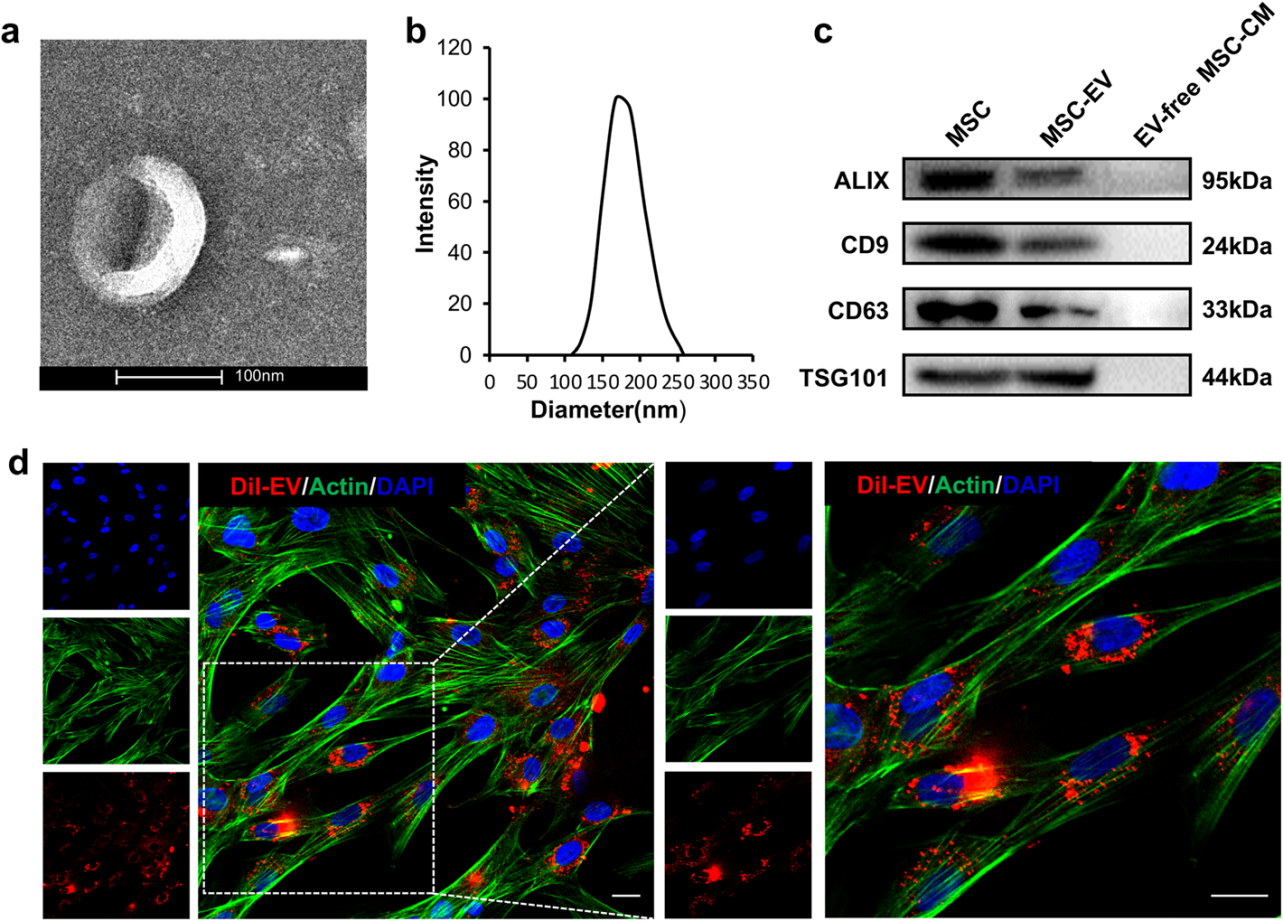
Characterization of hP-MSC-derived EVs. **a** TEM image of extracellular vesicles derived from hP-MSCs. Scale bars, 100 nm. **b** Particle size distribution of extracellular vesicles derived from hP-MSCs measured by dynamic light scattering (DLS). **c** The expression of ALIX, CD9, CD63, and TSG101, markers of extracellular vesicles derived from hP-MSCs. **d** The uptake of DiI-labeled (red) extracellular vesicles derived from hP-MSCs by hUC-MSCs (nucleus, blue). Scale bars, 20 μm

EVs used in this study were isolated from culture supernatant of hP-MSCs and characterized from the following aspects. The morphology was observed through TEM, and it was featured with typical round vesicle (Fig. 2a). Then, the particle size was further verified. As revealed in the dynamic light scattering analysis, the diameter of EV were about 100~200 nm, which was consistent with TEM image and supported by previous studies (Fig. 2b). Some proteins, Alix, CD9, CD63, and TSG101, were generally served as markers of EV, which were confirmed by western blotting (Fig. 2c). For the purpose of working efficiently, it was required for EVs to be internalized into targeted cells. To validate that, DiI-labeled EVs were incubated with hUC-MSCs for 6 h. The images that labeled EVs colocalized with nucleus or around them demonstrated the uptake of DiI-labeled EV by hUC-MSCs (Fig. 2d).

### MSC-EVs improved the cell viability

**Fig. 3:**
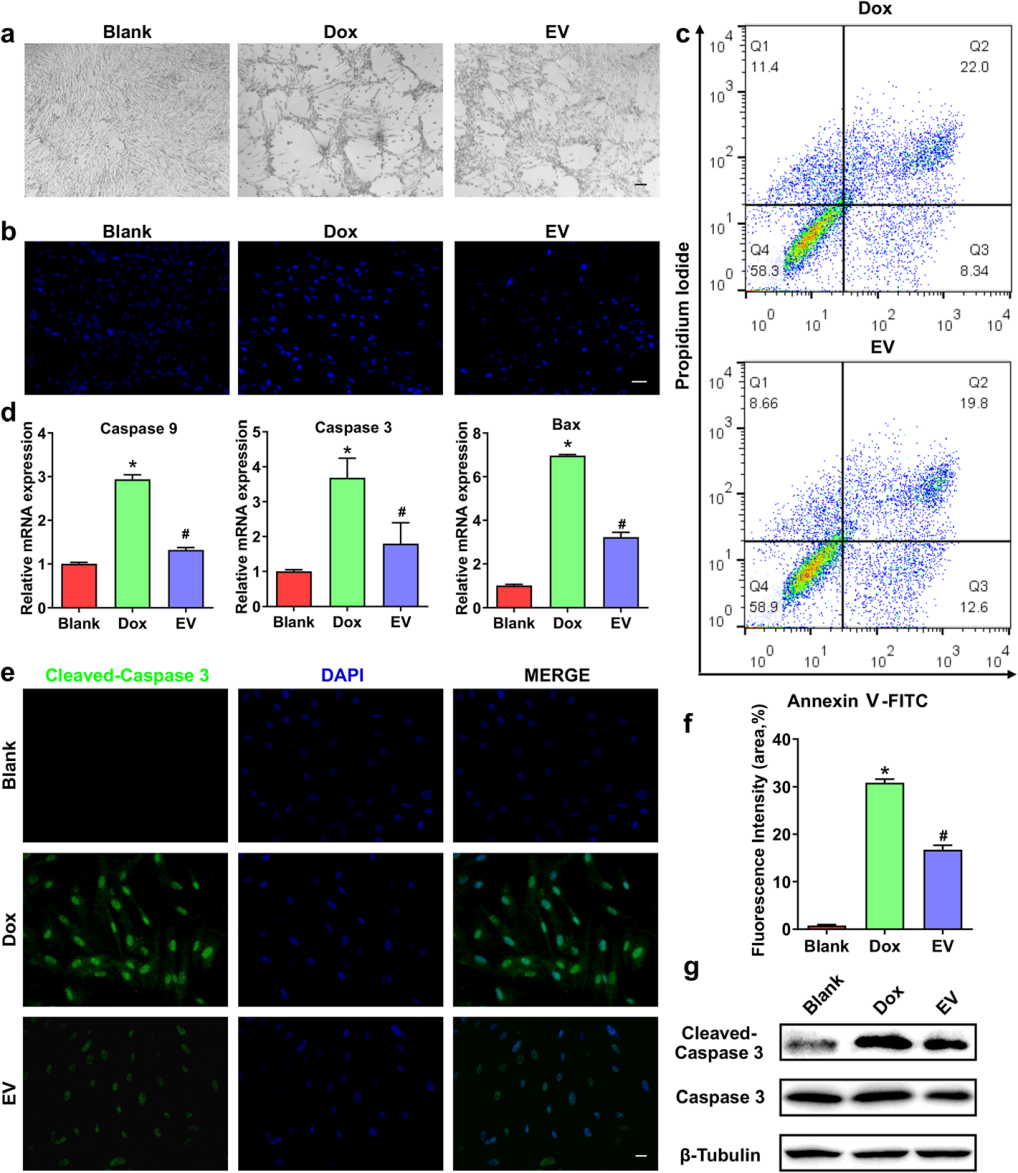
MSC-EVs improve the cell viability of Dox group. **a** The morphology of three groups (Blank, Dox, EV). Scale bars, 100 μm. **b** The apoptotic cells reflected by bright blue nuclei stained by Hoechst 33342. Scale bars, 50 μm. **c** Flow analysis of apoptotic cells in Dox and EV groups. The percent of apoptotic cells was detected by labeled Annexin V-FITC/PI. Annexin V^−^/PI^−^ represents living cells, Annexin V^+^/PI^−^ represents early apoptotic cells, Annexin V^−^/PI^+^ represents late apoptotic cells, and Annexin V^+^/PI^+^ represents dead cells. **d** mRNA expression of caspase 9, caspase 3, and bax. Relative gene expression was normalized to β-actin, and the data were analyzed via the 2^−ΔΔCt^ method. *n* = 3, ^*^P < 0.05 versus Blank groups; ^***#***^*P* < 0.05 versus Dox groups. **e** Representative immunofluorescence images of cleaved-caspase 3 (green) in hUC-MSCs treated with Dox and EV. Nuclei were counterstained with DAPI (blue). Scale bars, 20 μm. **f** Quantification of fluorescence intensity of cleaved-caspase 3 in hUC-MSCs with different treatment**.** *n* = 3, ^*^P < 0.05 versus Blank groups; ^***#***^*P* < 0.05 versus Dox groups. **g** The expression of caspase 3 and cleaved-caspase 3 detected by western blotting. All experiments were performed in three independent experiments and are shown as the mean ± SEM

The anti-apoptosis potential of MSC-derived extracellular vesicles in some biological processes have been widely studied, the therapeutic potential of which for cytotoxicity of chemotherapy is still unclear. In this study, we first explored the effects of hP-MSC-derived extracellular vesicles on doxorubicin-induced apoptosis. The cell morphology of three groups (Blank, Dox, EV) and the apoptosis reflected by Hoechst 33342 indicated that extracellular vesicles indeed relieved the cytotoxicity of doxorubicin (Fig. 3a, b). Then, flow analysis was to evaluate the cell viability of hUC-MSCs in three groups (Blank, Dox, EV). The early stage and late stage of apoptosis cells were stained by annexin V-FITC and propidium iodide (PI), respectively. Double positive (PI and annexin) cells were dead. Compared with Dox group, the apoptosis of hUC-MSCs in the EV group was significantly reduced (Fig. 3c). The mRNA expression of apoptosis-related factors, caspase 9, caspase3, and bax, were also downregulated in EV groups (Fig. 3d). Consistent with these results, in hUC-MSCs treated with doxorubicin, cleaved-caspase 3, a marker of apoptosis, was increased, indicating that doxorubicin induced apoptosis. Intriguingly, after treating hUC-MSCs with MSC-EVs (100 μg/ml) for 24 h, the fluorescence intensity of cleaved-caspase3 was reduced **(**Fig. 3e, f**)**. Further, we also performed the validation of caspase 3 and cleaved-caspase 3 proteins. From the results, the expression of caspase 3 is most in Dox group and reduced in EV group, which is almost consistent with above results. And, the expression of cleaved-caspase 3 also showed consistent tendency (Fig. 3g). In summary, it is definite that the cell apoptosis is decreased by hP-MSC-derived extracellular vesicles, and the cell viability is improved.

### Genome-wide analysis of miRNA expression in hUC-MSCs with different treatment

**Fig. 4:**
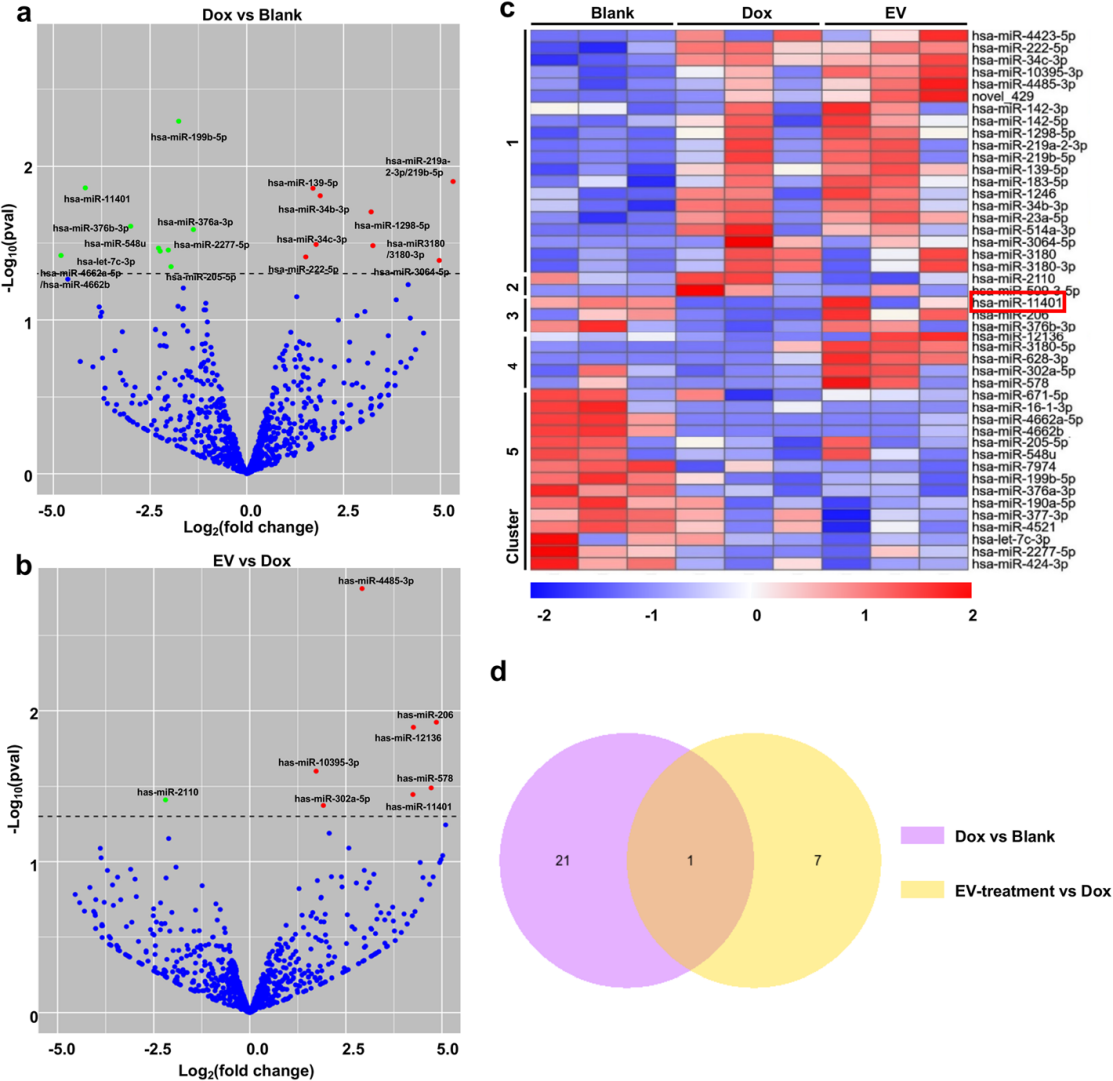
Genome-wide analysis of miRNA expression in UC-MSCs with different treatment. **a** The upregulated (red) and downregulated (green) miRNA in Dox groups compared to blank groups. **b** The upregulated (red) and downregulated (green) miRNA in EV groups compared to Dox groups. The comprehensive results are listed in Table 2. **c** The hierarchical cluster of miRNA in hUC-MSCs. The heatmap showed different miRNA expression pattern in three groups. Representative differential miRNA for each cell type are listed on the right side. The upregulation and downregulation of miRNA are labeled as red and blue, respectively. *n* = 3, *P* < 0.05. **d** The Venn diagram shows the overlapped miRNA among three groups

The above results indicated that the extracellular vesicles derived from hP-MSCs have anti-apoptosis effect. In order to investigate the anti-apoptosis mechanism, microRNA sequencing was performed to quantify the expression level of microRNAs in hUC-MSCs of different groups. In Dox group, there were averagely 12 upregulated and 10 downregulated miRNAs, in which the upregulation of hsa-miR-3180/3180-3p, hsa-miR-3064-5p, and has-miR-219b-5p and the downregulation of hsa-miR-199b-5p and hsa-miR-11401 were remarkable (Fig. 4a). With extracellular vesicle treatment for 24 h, 7 and 1 miRNA were upregulated and downregulated, respectively. Particularly, the expression of hsa-miR-11401 was upregulated (Fig. 4b). The differentiated expressed microRNAs were clustered, which was classified into 5 groups based on the expression pattern (Fig. 4c). In cluster 1, most miRNAs were concurrently upregulated in Dox and EV groups. Against the trend, most miRNA of cluster 5 were downregulated. In cluster 4, there was no great discrepancy between Blank and Dox groups, while the same miRNAs were upregulated in EV group. Because of the approximate expression of miRNA in Blank and EV groups, we focused on clusters 2 and 3. In EV groups, hsa-miR-RNA-2110 and hsa-miR-509-3-5p were downregulated, while hsa-miR-11401, hsa-miR-206, and hsa-miR-376b-3p were upregulated. The expression of these miRNAs were near to that of the Blank groups (Fig. 4c). Combining the volcano plot and cluster analysis, the downregulation of hsa-miR-11401 in Dox groups and its upregulation in EV groups were obviously labeled, which was revealed by a Venn diagram (Fig. 4d). That indicated the important role of hsa-miR-11401 in doxorubicin cytotoxicity, which may be one key factor that extracellular vesicles exerted protective effects.

**Fig. 5:**
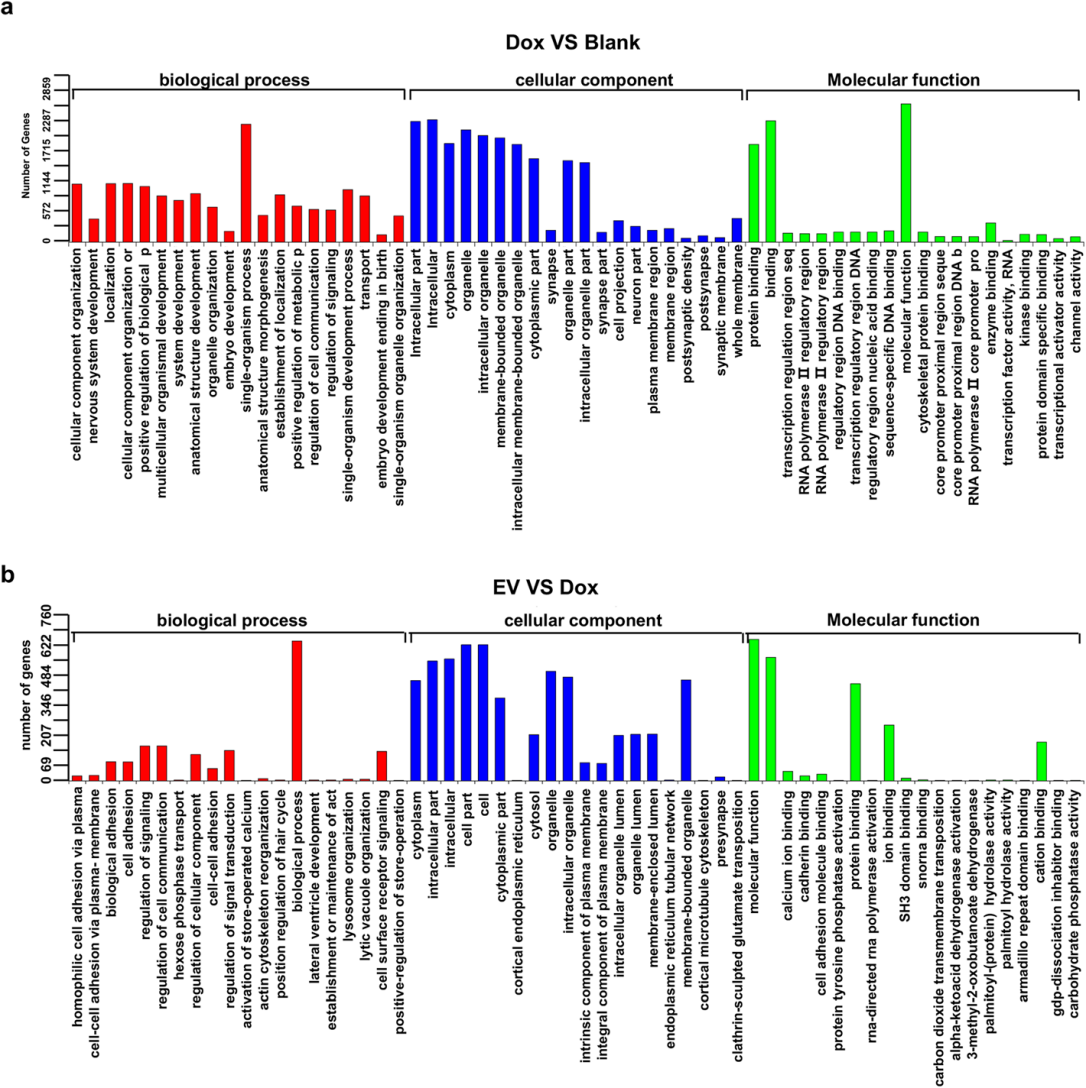
GO enrichment analysis of target genes. **a** The discrepancy of biological function, including biological process (BP), molecular function (MF), and cellular component (CC), between Blank and Dox groups. **b** The discrepancy of biological function, including biological process (BP), molecular function (MF), and cellular component (CC), between Dox and EV treatment groups. The distribution reflected the different biological function, the enriched target gene number of which was also different

Subsequently, the target genes of differentially expressed miRNAs mentioned were predicted with miRanda (http://www.microrna.org/microrna/home.do) and RNAhybrid (http://bibiserv.techfak.uni-bielefeld.de/rnahybrid/), two widely used miRNA bioinformation databases. These target genes were involved in a broad range of biological functions, the cellular processes, molecular function, and cellular component of which were markedly different. The distributions of enriched genes were also different in two groups, such as regulation of signaling and regulation of cell communication (Fig. 5a, b). The high-throughput miRNA sequencing and enrichment analysis of gene oncology indicated fundamental difference in miRNA expression and unveiled the discrepancy of biological functions in Dox and EV groups.

### Mechanism analysis of doxorubicin-induced hUC-MSC apoptosis

**Fig. 6:**
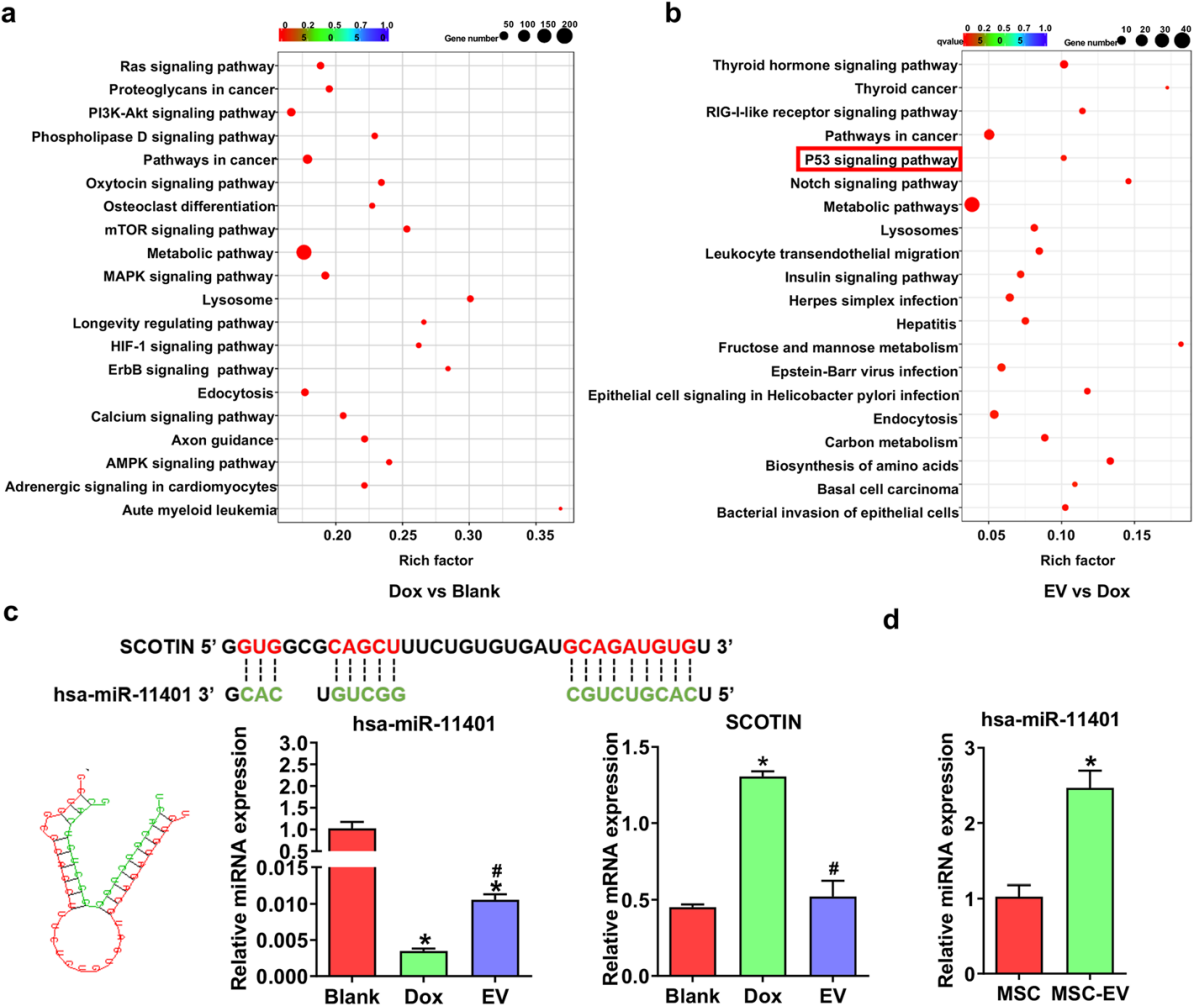
Mechanism analysis of doxorubicin-induced hUC-MSCs apoptosis. **a** Top 20 enriched pathways predicted by KOBAS based on the discrepancy of miRNA in Blank and Dox groups, which are related to the cytotoxicity of doxorubicin. **b** Top 20 enriched pathways predicted by KOBAS based on the discrepancy of miRNA in Dox and EV groups, which are related to the therapeutic potential of hP-MSC-derived extracellular vesicles for doxorubicin cytotoxicity. **c** The binding sites and match diagram between hsa-miR-11401 and SCOTIN are presented, and the expression of hsa-miR-11401 and SCOTIN among groups was further verified. The hsa-miR-11401 facilitated the downregulation of SCOTIN in EV groups. *n* = 3, ^*^P < 0.05 versus Blank groups; ^*#*^*P* < 0.05 versus Dox groups. **d** The expression of hsa-miR-11401 in extracellular vesicles showed that it can be delivered into recipient cells. *n* = 3,^*^P < 0.05 versus MSC

To further investigate the internal mechanisms on how extracellular vesicles inhibit cell apoptosis, pathway analysis of target genes was performed. From the results, some metabolic processes in Dox and EV groups were enriched, which indicated that doxorubicin disrupted the intracellular metabolism. Because of the anticancer effect of doxorubicin, some cancer-associated pathways were also enriched jointly. Apparently, the PI3K-Akt signaling pathway, MAPK signaling pathway, HIF-1α signaling pathway, and calcium signaling pathway were enriched in Dox groups (Fig. 6a), and the P53 signaling pathway was enriched in EV groups (Fig. 6b).

As mentioned above, hsa-miR-11401 was downregulated in Dox group and increased after EV treatment. Among the predicted target genes of hsa-miR-11401, SCOTIN (Shisa Family Member 5, SHISA5) is a protein related to DNA damage response [18]. It is the downstream modulator of P53 and can induce apoptosis through a “P53-Scotin-Cytc-Caspase9-Caspase3” manner. The binding sites between hsa-miR-11401 and SCOTIN are showed in Fig. 6c. Subsequently, the expression of hsa-miR-11401 in three groups were verified, which is consistent with results of microRNA sequencing. And, the mRNA expression of SCOTIN was also validated (Fig. 6c). The opposite trend to that of hsa-miR-11401 indicated that it certainly mediated the downregulation of SCOTIN and relieved apoptosis of hUC-MSCs in Dox groups, which also is supported by the reduced expression of caspase 3 in EV groups **(**Fig. 3e, g**)**. To explore whether the hsa-miR-11401 was delivered by extracellular vesicle, we also measured its expression in extracellular vesicle, which was highly expressed in EVs **(**Fig. 6d**)**. These results indicated that MSC-EVs transport hsa-miR-11401 to the target cells and relieve apoptosis.

### The low expression of hsa-miR-11401 inhibited its protective effects

**Fig. 7:**
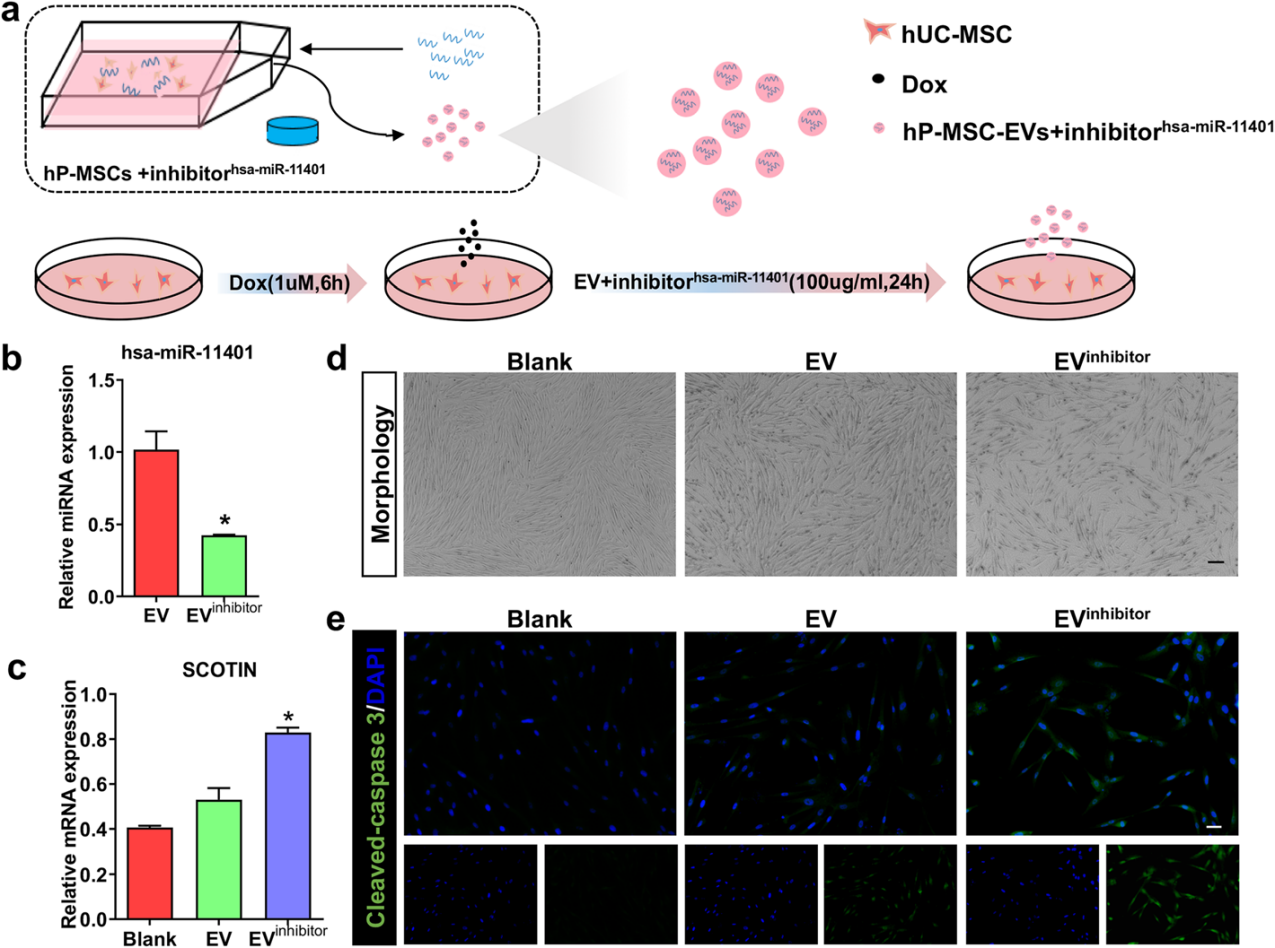
The low expression of hsa-miR-11401 inhibited its protective effects. **a** The mode pattern that collecting extracellular vesicles from inhibitor-pretreated hP-MSCs and the flow that EV^inhibitor^ repair the injured hUC-MSCs. **b** The expression of hsa-miR-11401 in EVs and inhibitor-contained EVs, ^*^P < 0.05 versus EV group. **c** The expression of SCOTIN in Blank, EV, and EV^inhibitor^ groups, ^*^P < 0.05 versus EV groups. **d** The morphology of hUC-MSCs in Blank, EV, and EV^inhibitor^ groups. Scale bars, 100 μm. **e** The expression of cleaved-caspase 3 in Blank, EV, and EV^inhibitor^ groups. Scale bars, 20 μm

To further validate the role of hsa-miR-11401 in relieving the cytotoxicity of doxorubicin, we designed the inhibitor of hsa-miR-11401 to abolish its expression in extracellular vesicles from hP-MSCs. As the model pattern showed (Fig. 7a), we collected the extracellular vesicles derived from inhibitor-pretreated hP-MSCs (EV^inhibitor^ group) to repair the damaged hUC-MSCs. First, we verified the low expression of hsa-miR-11401 in extracellular vesicles, which indicated that the inhibitor was successfully transfected into hP-MSCs (Fig. 7b). Then, the target gene, SCOTIN, was upregulated in EV^inhibitor^ group compared to that in the EV group (Fig. 7c). With extracellular vesicles treating damaged hUC-MSCs for 24 h, the cell intensity was reduced in EV^inhibitor^ group compared to that in the EV group (Fig. 7d), which indicated that the suppression of hsa-miR-11401 resulted in remarkably reduction in therapy effect of extracellular vesicles. Moreover, the expression of cleaved-caspase 3 was also quantified again (Fig. 7e). These results demonstrated that the hsa-miR-11401 in extracellular vesicles exerted great protective effect.

## Discussion

Chemotherapy is a forceful strategy for the therapy of various cancers, but the concomitant chemotherapy injury is the main obstacle restricting the use of chemotherapy drugs in clinic [19]. Mesenchymal stem cells (MSCs) play an important role in repair and regeneration of tissues, which also endow it the great remedy potential for chemotherapy injury, particularly the complications of doxorubicin cytotoxicity [7, 20–23]. It is important to sustain the function and viability of mesenchymal stem cells [24]. However, the mesenchymal stem cells may be injured by the nonspecific mechanism and duration of medication of chemotherapeutic agents [25, 26]. Therefore, the fate of mesenchymal stem cells during chemotherapy is not completely clear. Based on the remarkable adverse effects of doxorubicin and the therapy effect of MSCs on that [27, 28], doxorubicin was used to construct the in vitro cytotoxicity model in this study. The effect of MSC-derived extracellular vesicles for chemotherapy-induced cytotoxicity was evaluated, which was demonstrated by the in vitro cytotoxicity model of doxorubicin pretreating cells, followed by treatment with MSC-derived extracellular vesicles. The model was also used to verify whether injured MSCs can be repaired by extracellular vesicles from other MSCs, which can contribute to the self-healing of MSCs in vivo and enhance their protective effect in vitro. Our results showed that doxorubicin accumulation in cells is increasing in a time-dependent manner. Simultaneously, the cell vitality was inversely decreasing, which was evaluated by the high expression of cleaved-caspase 3 and percent of apoptotic cells. MSC-derived extracellular vesicles significantly relieved cell apoptosis and improved cell survival, suggesting that the paracrine is the main mechanism of MSCs to exert protective effect.

**Fig. 8:**
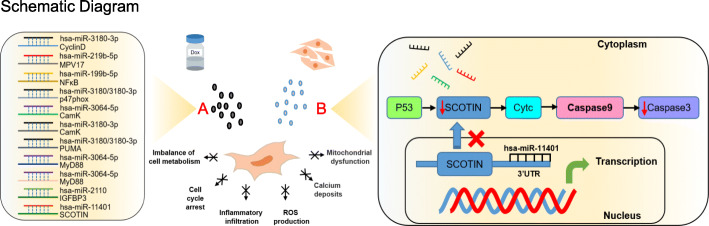
The schematic diagram that doxorubicin cytotoxicity induced by the remarkably differentially expressed miRNA and the protective mechanism of extracellular vesicles from hP-MSCs. The cytotoxicity of doxorubicin for hUC-MSCs is severe. The treated hUC-MSCs showed great discrepancy compared to normal hUC-MSCs, which was supported by the results of miRNA sequencing. Among the differentially expressed miRNA, some were remarkably labeled, the target gene prediction of which indicated that the adverse effects, cell cycle arrest, inflammatory infiltration, ROS production, calcium deposit, and mitochondrial dysfunction, may be involved the cytotoxicity of doxorubicin and result in apoptosis. In this process, the extracellular vesicles from hP-MSC can exert protective effect through the downregulation of SCOTIN mediated by hsa-miR-11401 and reduce the expression of cleaved-caspase 3, alleviating the apoptosis induced by doxorubicin

MiRNA sequencing revealed the internal discrepancy among three groups. Particularly, hsa-miR-11401 was showed having great reduction in hUC-MSCs of Dox groups. And extracellular vesicles upregulated it near to that of blank groups. Combining the enriched pathways in EV groups, hsa-miR-11401 and the p53 signaling pathway were taken for further research. SCOTIN, the target gene of hsa-miR-11401, is related to DNA damage response and can induce apoptosis [18, 29, 30]. As reported in previous studies, p53 is a transcription factor that leads to growth arrest and apoptosis, the relative downstream genes of which are complicated [18, 31–33]. SCOTIN is a p53-inducible proapoptotic gene and the downregulation of SCOTIN can increase the intracellular anti-apoptosis property, which indicated that the great significance of SCOTIN in p53-dependent apoptosis [18, 34–36]. The KEGG network of p53 signaling pathway also indicated the SOCTIN induce apoptosis through “p53-Scotin-Cytc-Caspase9-Caspase3” signaling pathway. We validated the expression of SCOTIN among three groups. As expected, extracellular vesicles certainly downregulated the expression. That manifested DNA damage may be one of the main manners that doxorubicin inducing cytotoxicity and SCOTIN can be taken as a target for the exploration of therapeutic strategies [37–39]. Moreover, the high expression of hsa-miR-11401 in extracellular vesicle proved that it can be delivered into recipient cells and confirmed the feasibility in applying extracellular vesicle for doxorubicin cytotoxicity, which was further supported by inhibiting the expression of hsa-miR-11401 in extracellular vesicles (Fig. 8). Additionally, in Dox groups, the expression of hsa-miR-3180/3180-3p, hsa-miR-3064-5p, and hsa-miR-219b-5p were upregulated and hsa-miR-199b-5p were downregulated compared to normal hUC-MSCs. And the downregulation of hsa-miR-2110 in EV groups was obviously labeled. The target gene prediction of these miRNA and enrichment analysis of pathway indicated that the apoptosis of hUC-MSC in Dox group was associated with cell cycle arrest, inflammatory infiltration, ROS production, calcium deposit, and mitochondrial dysfunction [40–44]. These pathological phenomena can partly explain the complex mechanisms involved in the doxorubicin cytotoxicity and are waiting for further verification.

In summary, the therapeutic potential of MSC-derived extracellular vesicles for the apoptosis of hUC-MSCs during chemotherapy is verified in this study. Moreover, because anticancer is the main effect of doxorubicin and MSCs have great tolerance property than terminally differentiated cell, with doxorubicin killing cancer cells and being completely removed, employing the MSC-derived extracellular vesicles to remedy damaged MSCs may not interfere the curative effect of doxorubicin for cancer. Moreover, the miRNA screened in miRNA sequencing and the relative mechanism can be taken as targets for further studies. Particularly, hsa-miR-11401 and its target protein, SCOTIN, can provide some thoughts for the therapy of doxorubicin cytotoxicity.

## Conclusions

Our study revealed that accumulation of doxorubicin in hUC-MSCs decreased cell viability in a time-dependent manner, which is similar to cancer cells reported by previous studies. Intriguingly, the hP-MSC-derived extracellular vesicles relieved cell apoptosis and improved cell survival rate. MiRNA sequencing presented different miRNA expression pattern of normal and doxorubicin-treated hUC-MSCs. Hsa-miR-11401 was identified to mediate the downregulation of SCOTIN and blocked the p53-dependent apoptosis. Pathway analysis of miRNA target genes also implied the toxic effect of doxorubicin for hUC-MSCs is associated with cell cycle arrest, inflammatory infiltration, ROS production, calcium deposit, and mitochondrial dysfunction. In conclusion, the inherent effects involved in doxorubicin cytotoxicity were complex, which contributed to hUC-MSC apoptosis. The application of extracellular vesicles blocked this process and the possibility of hUC-MSC self-healing during chemotherapy is verified.

## Supplementary Information

**Additional file 1.**

## Data Availability

The dataset used and/or analyzed during the current study are available from the corresponding author upon reasonable request.

## Abbreviations

MSCs: Mesenchymal stem cells
EVs: Extracellular vesicles
hUC-MSCs: Human umbilical cord-derived mesenchymal stem cells
hP-MSCs: Human placental mesenchymal stem cells
Dox: Doxorubicin
SHISA5: Shisa Family Member 5
DMEM: Dulbecco’s modified Eagle’s medium
PBS: Phosphate-buffered saline
TEM: Transmission electron microscopy
DLS: Dynamic light scattering
RIPA: Radio-immunoprecipitation assay buffer
PVDF: Polyvinylidene fluoride
CCK-8: Cell counting kit-8
DAPI: 4,6-Diamidino-2-phenylindole
GO: Gene ontology
KEGG: Kyoto Encyclopedia of Genes and Genomes
