# Supplementary material for: The delivery of hsa-miR-11401 by extracellular vesicles can relieve doxorubicin-induced mesenchymal stem cell apoptosis
Source: Stem Cell Res Ther. 2021 Jan 22;12:77. doi: 10.1186/s13287-021-02156-5 (PMC7821514; doi:10.1186/s13287-021-02156-5)
Supplement: Supplementary file 1 — Additional file 1. [file 13287_2021_2156_MOESM1_ESM.docx]

**Supplemental Information:**

**The delivery of hsa-miR-11401 by extracellular vesicles can relieve doxorubicin-induced mesenchymal stem cell apoptosis**

**Li *et al.***

Supplemental figures:

Figure S-1: The binding sites and match diagram between different miRNA and mRNA.

Figure S-2: The expression of protein markers of extracellular vesicle in MSC, EV-free FBS, EV-free cell conditioned medium from hP-MSC, MSC-EVs and DMEM/F12 medium containing 10% EV-free FBS.

Supplemental tables:

Table 1. The sequences of primers used in this study;

Table 2. The different expression of miRNA among three groups (Blank, Dox, EV).

**
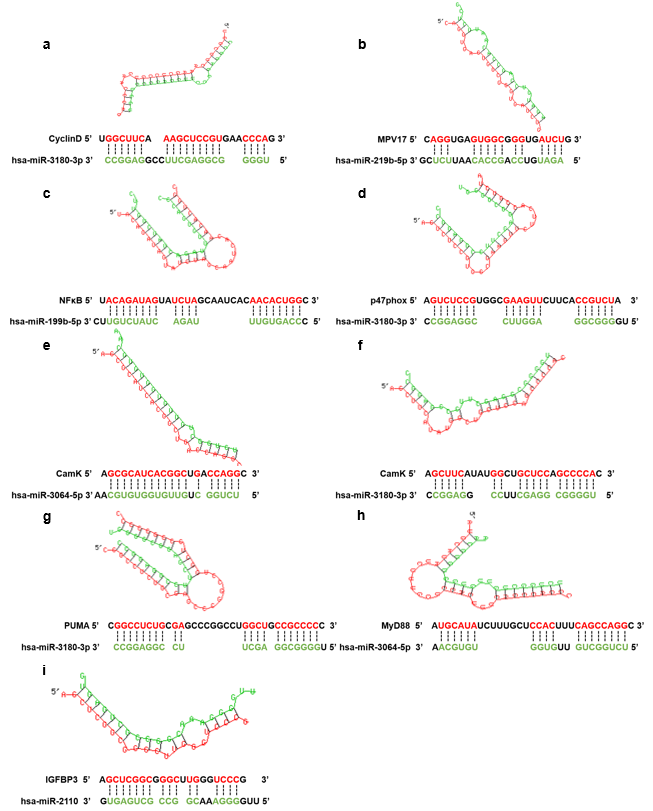
**

**Figure S-1: The binding sites and match diagram between different miRNA and mRNA. (a)** The combination of hsa-miR-3180-3p and CyclinD, which regulate the cell cycle. The downregulation of CyclinD induces cell cycle arrest. **(b)** The combination of has-miR-219b-5p and MPV17. **(c)** The match sites of has-199b-5p and NFκB, key protein mediating inflammation. The downregulation of NFκB facilitates the inflammatory infiltration. **(d)** The combination of hsa-miR-3180/3180-3p and p47phox. MPV17 and p47phox are is related to ROS metabolism. **(e)** and **(f)** The match sites between hsa-miR-3180/3180-3p and CamK, which mediated the downregulation of CamK, leading to calcium deposits. **(g)** The combination of PUMA and hsa-miR-3180/3180-3p, **(h)** Myd88 and hsa-miR-3064-5p, and **(i)** IGFBP and hsa-miR-2110, which are related to mitochondrial dysfunction. The binding sites and match diagram were analyzed by RNA hybrid.

**
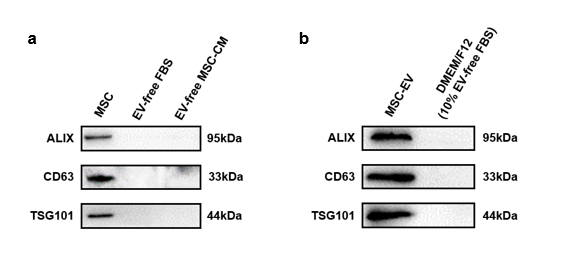
**

**Figure S-2: The expression of protein markers of extracellular vesicle in MSC, EV-free FBS, EV-free cell conditioned medium from hP-MSC, MSC-EVs and DMEM/F12 medium containing 10% EV-free FBS. (a)** The expression of ALIX, CD63 and TSG101 in MSCs, EV-free FBS and EV-free cell conditioned medium from hP-MSCs. **(b)** The expression of ALIX, CD63 and TSG101 in MSC-EVs and DMEM/F12 medium containing 10% EV-free FBS. In detail, the medium was ultracentrifuged according to the protocol that isolate EVs from cell supernatant of hP-MSCs used in our study.

Table 1. The sequences of primers used in this study

| **Genes** | **Forward primer** | **Reverse prime** |
| --- | --- | --- |
| β-actin | CTCCATCCTGGCCTCGCTGT | GCTGTCACCTTCACCGTTCC |
| Caspase 3 | GTTTGAGGACCTTCGACCAG | CAAAGATGTCGTCCAGGGTC |
| Caspase 9 | TGAACTTCTGCCGTGAGTCC | TCCCTTTCACCGAAACAGCA |
| Bax | TCCACCAAGAAGCTGAGCGAG | GTCCAGCCCATGATGGTTCT |
| Scotin | CAAAGATGTCGTCCAGGGTC | ATCACTGAGGCTGTGGCGGCACTGC |
| has-miR-11401 | GCCGAGTCACGTCTGCGGCT | CTCAACTGGTGTCGTGGAGTCG |
| has-miR-11401 RT | CTCAACTGGTGTCGTGGAGTCGGCAATTCAGTTGAGGTCACG | |

Table 2. The different expression of miRNA among three groups (Blank, Dox, EV)

| **Dox vs Blank** | | | | | | |
| --- | --- | --- | --- | --- | --- | --- |
|  |  |  |  |  |  |  |
| **miRNA** | | **Blank-readcount** | **Dox-readcount** | **log2FoldChange** | **pval** | **padj** |
| Downregulation | hsa-miR-199b-5p | 3174.529 | 936.717 | -1.7606 | 0.005087 | 0.9986 |
|  | hsa-miR-11401 | 10.80157 | 0.591257 | -4.1708 | 0.013815 | 0.9986 |
|  | hsa-miR-376b-3p | 16.92858 | 2.076414 | -3.001 | 0.024567 | 0.9986 |
|  | hsa-miR-376a-3p | 183.7393 | 70.67824 | -1.3803 | 0.025821 | 0.9986 |
|  | hsa-miR-548u | 29.38202 | 6.03707 | -2.2816 | 0.034124 | 0.9986 |
|  | hsa-miR-2277-5p | 25.47569 | 6.292149 | -2.029 | 0.035137 | 0.9986 |
|  | hsa-let-7c-3p | 56.32313 | 11.99874 | -2.2407 | 0.035709 | 0.9986 |
|  | hsa-miR-4662a-5p | 4.646004 | 0 | -4.7982 | 0.0381 | 0.9986 |
|  | hsa-miR-4662b | 4.646004 | 0 | -4.7982 | 0.0381 | 0.9986 |
| Upregulation | hsa-miR-219a-2-3p | 0.549388 | 18.31768 | 5.5914 | 0.001627 | 0.9986 |
|  | hsa-miR-219b-5p | 0.549388 | 18.31768 | 5.5914 | 0.001627 | 0.9986 |
|  | hsa-miR-514a-3p | 0 | 11.2598 | 5.8557 | 0.005399 | 0.9986 |
|  | hsa-miR-509-3-5p | 0 | 7.804707 | 5.3276 | 0.012554 | 0.9986 |
|  | hsa-miR-139-5p | 54.71744 | 177.2829 | 1.7082 | 0.013902 | 0.9986 |
|  | hsa-miR-34b-3p | 39.44259 | 147.2114 | 1.8956 | 0.015552 | 0.9986 |
|  | hsa-miR-1298-5p | 2.291407 | 19.18089 | 3.2098 | 0.019804 | 0.9986 |
|  | hsa-miR-34c-3p | 14.1665 | 48.72255 | 1.7889 | 0.032264 | 0.9986 |
|  | hsa-miR-3180 | 1.420398 | 11.73358 | 3.2551 | 0.03286 | 0.9986 |
|  | hsa-miR-3180-3p | 1.420398 | 11.73358 | 3.2551 | 0.03286 | 0.9986 |
|  | hsa-miR-222-5p | 40.63553 | 117.2812 | 1.5225 | 0.038944 | 0.9986 |
|  |  |  |  |  |  |  |
| **EV vs Dox** | | | | | | |
|  |  |  |  |  |  |  |
| **sRNA** | | **Dox-readcount** | **EV-readcount** | **log2FoldChange** | **pval** | **padj** |
| Downregulation | hsa-miR-2110 | 28.36545 | 6.184539 | -2.1938 | 0.03886 | 0.99988 |
| Upregulation | hsa-miR-4485-3p | 25.02369 | 189.6553 | 2.9219 | 0.001544 | 0.99988 |
|  | hsa-miR-206 | 0.318455 | 10.25655 | 4.8553 | 0.011934 | 0.99988 |
|  | hsa-miR-12136 | 0.982119 | 18.85331 | 4.2575 | 0.012888 | 0.99988 |
|  | hsa-miR-10395-3p | 24.63198 | 81.46302 | 1.7256 | 0.025061 | 0.99988 |
|  | hsa-miR-578 | 0.345209 | 9.349961 | 4.7207 | 0.032358 | 0.99988 |
|  | hsa-miR-11401 | 0.690418 | 12.83263 | 4.2449 | 0.035795 | 0.99988 |
|  | hsa-miR-302a-5p | 10.38259 | 39.07213 | 1.9153 | 0.042307 | 0.99988 |
